# Evaluating diagnostic content of AI-generated chest radiography: A multi-center visual Turing test

**DOI:** 10.1371/journal.pone.0279349

**Published:** 2023-04-12

**Authors:** Youho Myong, Dan Yoon, Byeong Soo Kim, Young Gyun Kim, Yongsik Sim, Suji Lee, Jiyoung Yoon, Minwoo Cho, Sungwan Kim

**Affiliations:** 1 Department of Biomedical Engineering, Seoul National University College of Medicine, Seoul, Republic of Korea; 2 Department of Rehabilitation Medicine, Seoul National University Hospital, Seoul, Republic of Korea; 3 Interdisciplinary Program in Bioengineering, Seoul National University Graduate School, Seoul, Republic of Korea; 4 Department of Radiology, Severance Hospital, Yonsei University College of Medicine, Seoul, Republic of Korea; 5 Department of Radiology and Research Institute of Radiological Science, Yonsei University College of Medicine, Seoul, Republic of Korea; 6 Transdisciplinary Department of Medicine and Advanced Technology, Seoul National University Hospital, Seoul, Republic of Korea; 7 Medical Big Data Research Center, Seoul National University College of Medicine, Seoul, Republic of Korea; 8 Biomedical Research Institute, Seoul National University Hospital, Seoul, Republic of Korea; 9 Institute of Bioengineering, Seoul National University, Seoul, Republic of Korea; Xiamen University - Malaysia, MALAYSIA

## Abstract

**Background:**

Accurate interpretation of chest radiographs requires years of medical training, and many countries face a shortage of medical professionals to meet such requirements. Recent advancements in artificial intelligence (AI) have aided diagnoses; however, their performance is often limited due to data imbalance. The aim of this study was to augment imbalanced medical data using generative adversarial networks (GANs) and evaluate the clinical quality of the generated images via a multi-center visual Turing test.

**Methods:**

Using six chest radiograph datasets, (MIMIC, CheXPert, CXR8, JSRT, VBD, and OpenI), starGAN v2 generated chest radiographs with specific pathologies. Five board-certified radiologists from three university hospitals, each with at least five years of clinical experience, evaluated the image quality through a visual Turing test. Further evaluations were performed to investigate whether GAN augmentation enhanced the convolutional neural network (CNN) classifier performances.

**Results:**

In terms of identifying GAN images as artificial, there was no significant difference in the sensitivity between radiologists and random guessing (result of radiologists: 147/275 (53.5%) vs result of random guessing: 137.5/275, (50%); p = .284). GAN augmentation enhanced CNN classifier performance by 11.7%.

**Conclusion:**

Radiologists effectively classified chest pathologies with synthesized radiographs, suggesting that the images contained adequate clinical information. Furthermore, GAN augmentation enhanced CNN performance, providing a bypass to overcome data imbalance in medical AI training. CNN based methods rely on the amount and quality of training data; the present study showed that GAN augmentation could effectively augment training data for medical AI.

## 1. Introduction

Chest radiography is one of the most widely used medical imaging modalities in the United States [1] as it is affordable and quick, and can rule out several critical pathological conditions such as COVID-19 pneumonia [2–5], lung cancer [6–8], pulmonary tuberculosis [9], and heart diseases [10]. In South Korea, the number of chest radiograph prescriptions has steadily increased from 23 million per year in 2015 to almost 27 million in 2019 [11].

The clinical benefit of chest radiography is user dependent [12]. Chest radiography holds a vast amount of information in a single image, which requires extensive medical training for accurate interpretation. Training a radiologist requires years, and the number of experts who can read chest radiographs is not increasing as rapidly as the number of prescriptions. In many economies, this gap in the supply and demand of medical professionals is worse in medically underserved rural areas [12–14]. Due to urban migration, these areas often have elderly populations, who are at an elevated risk of cardiovascular and pulmonary maladies that can be screened through chest radiographs, However, due to an imbalance in the distribution of healthcare resources, such clinical demands are rarely met. Medical artificial intelligence (AI) can address such shortages of clinical expertise if it can be trained to achieve levels of performance comparable to experienced physicians in interpreting specific diagnostic modalities [1]. With appropriate development and distribution of medical AI as computer-aided diagnosis programs, the unmet needs for clinical expertise in medically underserved areas can be better addressed [15].

With the rapid advancement of artificial intelligence, computer-aided diagnosis research has recently bloomed [1, 11]. The same is true with the development of deep learning, which has resulted in remarkable progress being made in the field of medical image analysis [16]. One of the predominant challenges to AI training in the medical imaging is data imbalance. To ensure that the AI model is robust and effective, the training dataset must be extensive and well balanced [17]; however, the amount of medical data is usually insufficient and the imbalance, large [18]. In many cases, the most pernicious diseases have the lowest prevalence, leading to severe data imbalances [19], which can negatively affect model performances.

Many chest radiograph datasets are publicly available, and most are prone to data imbalance. Chest X-ray 8 (CXR8), one of the largest existing open-source chest radiograph data initiatives led by the National Institute of Health, is no exception; of the 108,948 chest anteroposterior radiographs in the dataset, 84,312 (77.39%) images had no pathologic lesions, while only 1,062 (0.97%) images showed signs of pneumonia [20]. MIMIC-CXR, another extensive chest radiograph dataset created by the MIT Laboratory for Computational Physiology and Beth Israel Deaconess Medical Center, comprises 377,110 images. Of these images, only 10,801 (2.86%) contained pulmonary masses and 25,038 (6.64%) contained evidence of pneumonia [21].

With the evolution of medical AI research, many data augmentation methods have been developed to overcome such data imbalances [18]. In computer vision, common traditional data augmentation methods include flipping, rotating, shifting, and color-transforming images to oversample underrepresented data [17, 22], while in some medical images, including chest radiographs, traditional augmentation has been proven to result in worse model performance [11]. To overcome this, many novel data augmentation techniques have been developed including generative adversarial networks (GAN).

GAN is a powerful method to generate novel images without supervision [16, 23]. The algorithm employs two competing ‘adversarial’ networks: generator network G(z) and discriminator network D(x) [24]. G(z) produces realistic images and attempts to deceive D(x), while D(x) learns to better discriminate between real and fake images [25]. While G(z) seeks to minimize the cost value function V(D, G), D(x) seeks to maximize it. Consequently, GAN learns to create new images similar to the original ones.

Recently, a new type of GAN, conditional GAN (cGAN), was introduced [18]. cGAN can produce guided images with specific features, using a conditional latent variable to guide the image generation of a designated component [26]. For example, cGAN enables researchers to produce chest radiographs with anomalies from normal images. Therefore, with cGAN, it is now possible to generate images of pathologies with low incidence, thereby augmenting the dataset such that rare diseases are equally represented. The idea of conditional image generation has also been applied in image-to-image translation. By providing conditional domain information, the input image can be translated to various target domains, and the network can learn the relevant features of multiple domains through a single model.

In this study, a repertoire of realistic chest radiographs was synthesized using image-to-image translation; subsequently, the clinical competency of generated images was assessed through multi-center visual Turing test by five board-certified radiologists from three university hospitals. Furthermore, an investigation was conducted to discern whether GAN augmentation enhances convolutional neural network (CNN) performances by evaluating three CNN classifiers trained on the original and GAN-augmented datasets. Therefore, the aim of this study was to augment imbalanced medical data using generative adversarial networks and evaluate the clinical quality of the generated images via a multi-center visual Turing test.

## 2. Methods

This study was HIPAA compliant. Per the Office for Human Research Protections (45CFR46.102), publicly available data do not require Institutional Review Board (IRB) review. Nevertheless, Seoul National University Hospital IRB reviewed the study protocol and the need for informed consent was waived (IRB number E-2211-041-1375).

This study comprised two major tasks: first, publicly available datasets were used to train GAN to synthesize realistic chest radiographs; and second, the generated images were evaluated through 1) a multi-center visual Turing test and 2) the performance metrics of the CNN-based classifier trained on original and GAN-augmented dataset.

### 2.1 Image synthesis

#### 2.1.1 Dataset

Six publicly available chest radiograph datasets were used for both the GAN and CNN-based classifiers, namely ChestX-ray 8, MIMIC-CXR, CheXpert, JSRT, VBD, and OpenI. The details of the datasets used in this study are presented below in Table 1.

**Table 1:** Details of datasets included in this study.

Name	Institution	# of Images	Geographic Region	Year
ChestX-ray8 [20]	NIH	112,120	Northeast USA	2017
CheXpert [27]	Stanford University	223,414	Western USA	2019
MIMIC-CXR [21]	MIT	377,095	Northeast USA	2019
JSRT [28]	JSRT	247	Japan	2000
VinDr-CXR [29]	VinBrain Group	15,000	Vietnam	2020
OpenI [30]	Indiana University	7,470	Northeast USA	2016

Of these six datasets, one (OpenI) was reserved to construct a separate test set, while the other five were used to construct the training/validation set to train the GAN and CNN-based classifiers. To represent cardiac, pulmonary, and neoplastic pathologies, pleural effusion (pulmonary), cardiomegaly (cardiac), and lung mass (neoplastic) were used as disease labels. Images with unique labels (only one positive disease label) were obtained. The distribution of labels in the primary repertoire of raw data is presented in Table 2.

**Table 2:** Distribution of labels of included datasets.

	CXR8	MIMIC	CheXPert	JSRT	VBD	OpenI	Total Labels
Mass	755	220	1,839	154	289	97	3,354
Effusion	2,111	966	819	-	302	96	4,294
Cardiomegaly	3,011	363	327	-	1,112	297	5,110
No Findings	59,891	4,042	2,502	93	10,607	1,379	78,514
**Total Images**	65,768	5,591	5,487	247	12,310	1,869	91,272

The cumulative training/validation dataset consisted of 1,560 radiographs in the lung mass class, 672 in the effusion class, 260 in the cardiomegaly class, and 3,230 in the no pathological findings class. The separate test set, extracted from the OpenI dataset, consisted of 97 in the lung mass class, 96 in the effusion class, 297 in the cardiomegaly class, and 1,379 in the no pathological findings class. The distribution of labels in the training/validation/test sets is presented in Table 3.

**Table 3:** Distribution of labels of the train/validation/test set.

	Train/Validation (%)	Augmented Train/Validation (%)	Test (%)
Lung Mass	1,560 (27.3)	3,230 (25.0)	83 (5.2)
Effusion	672 (11.7)	3,230 (25.0)	71 (4.4)
Cardiomegaly	260 (4.5)	3,230 (25.0)	212 (13.3)
No Findings	3,230 (56.4)	3,230 (25.0)	1,232 (77.1)
**Total**	5,722 (100.0)	12,920 (100.0)	1,598 (100.0)

#### 2.1.2 Data pre-processing

**Fig 1:**
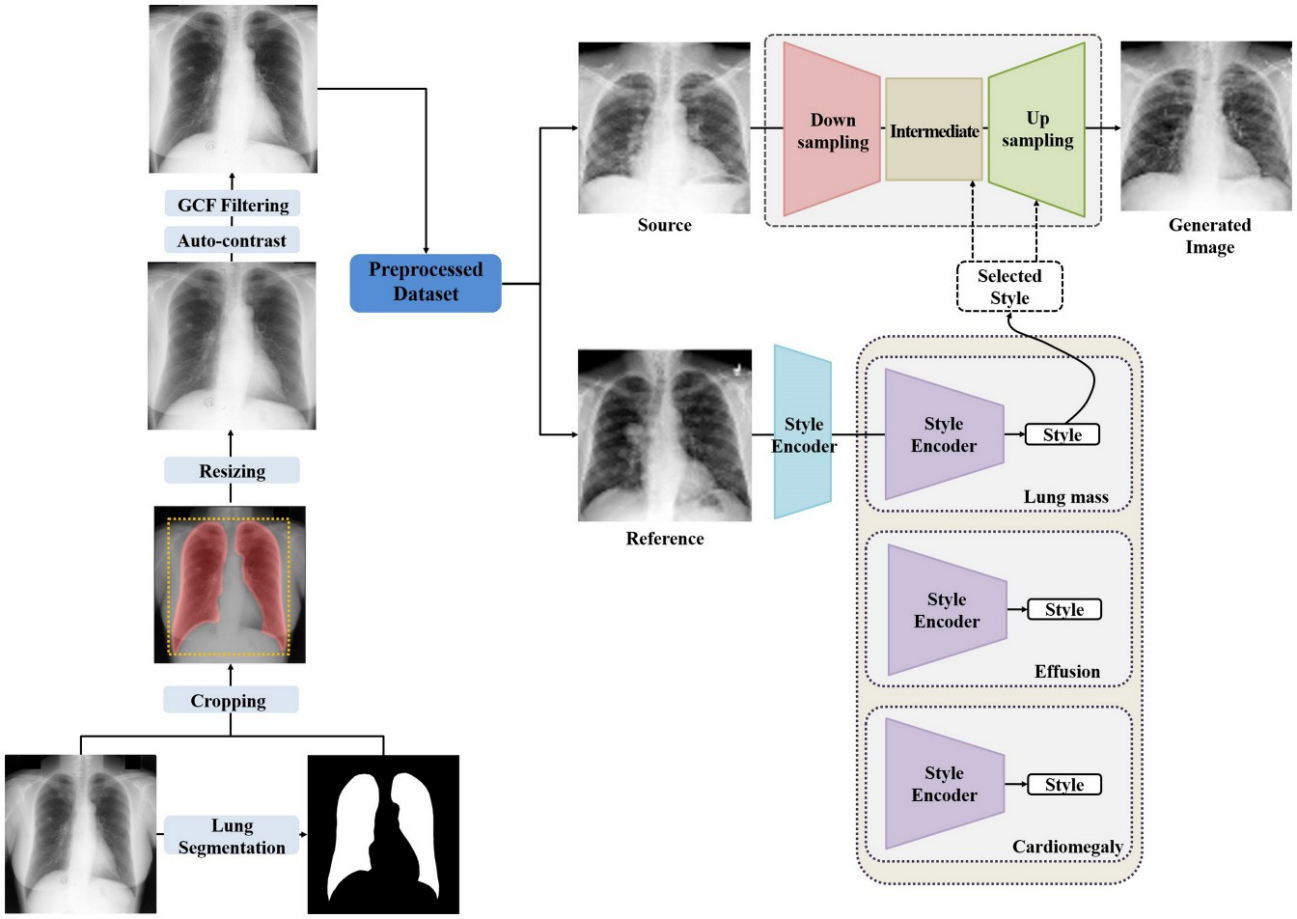
Chest radiography synthesis workflow.

Using in-house U-Net-based lung segmentation software, each image in the cumulative dataset was processed to yield a square region of interest that contained lung fields only. The cropped images were converted to 256 by 256 pixels and auto-contrasted after which they were filtered with global contrast factor (GCF) threshold before training the GAN- and CNN-based classifiers. This process is illustrated in Fig 1.

#### 2.1.3 Traditional augmentation

To construct the first of the two augmented datasets, the original cumulative dataset was augmented using traditional augmentation methods, such as geometric rotation, flipping, cropping, and scaling. The augmentation process included random rotation between −10 and 10 degrees, random linear translation between −10 and 10 pixels, horizontal flipping, random scaling between factors 0.85 and 1.15, and random contrast between 90% and 110%. The conventional augmentation schemes employed in this study are widely used in computer vision research [17], yet have been reported to result in overfitting [19] or computational burden without performance enhancement [22]. Traditional augmentation has been reported to occasionally result in worse performance metrics [22]. The dataset was augmented such that the underrepresented positive label classes would be balanced with the ‘no findings’ class. The data distribution for the augmented sets is presented in Table 3.

#### 2.1.4 GAN augmentation

GAN employ two neural networks to create plausible synthetic data that retains the critical features of the original data [23]. Recent developments in GAN research have resulted in impressive levels of image generation using image-to-image translation. In image-to-image translation, the network learns to map a feature from input x onto a target output y; therefore, one can expect to generate an image with the desired disease label from a normal input image [26]. In this study, a unified conditional GAN, StarGAN v2, was used to perform GAN augmentation.

**Fig 2:**
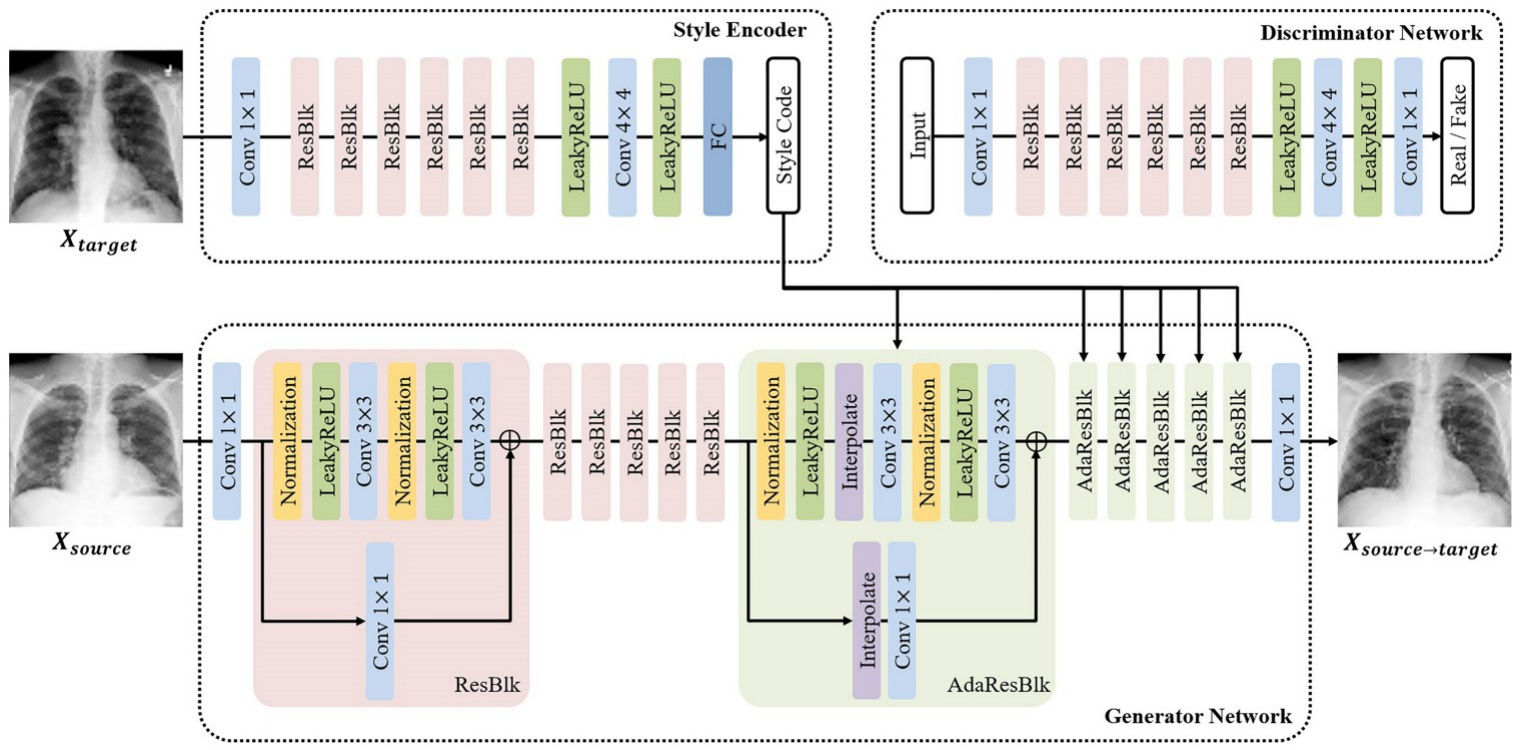
Generative adversarial networks architecture.

StarGAN v2 was trained to create (i) a cardiomegaly class from the normal class, (ii) an effusion class from the normal class, and (iii) a lung mass class from the normal class. As shown in Fig 2, the image styles of each class were transferred using instance normalization layers managed by the conditional encoder. The object structure and texture information were analyzed separately to generate the combined images after which the geometric information of the object was encoded using ResNet-like encoders (ResBlk) while low-level features of the texture information were extracted using style encoders. Subsequently, each AdaInResBlk block containing AdaIn modules modulated the learned geometric representation by cues received from the style encoder. To quantitatively evaluate the quality of the generated images, the differences between the two distributions in the high-dimensional feature space of an InceptionV3 classifier with a Fréchet inception distance score were measured. The augmentation process is illustrated in Fig 2, whereas the data distribution for the augmented set is presented in Table 3.

### 2.2 Experiments

#### 2.2.1 Visual Turing test

Five board-certified radiologists from three different university hospitals, each with at least five years of clinical experience, independently assessed the quality of GAN-generated chest radiographs. Each radiologist was given a set of 103 chest radiographs (55 GAN-generated and 48 real) and had to decide whether each image was real or artificial using visual analysis without a time limit. Each radiologist was blinded to the composition of the test set. The mean accuracy, sensitivity, specificity, F1-score, and Matthews correlation coefficient (MCC) of the five experts were calculated. Mann-Whitney U test was used to evaluate whether the mean of the expert performance was better than random guessing. McNemar test was used to investigate whether each expert response was significantly different from random.

To assess whether the GAN-generated images were not only realistic but also clinically informative, the radiologists were further assigned to two classification tasks. For the first task, the radiologists classified 103 actual chest radiographs into four classes: effusion, cardiomegaly, lung mass, and no findings. The second task was identical to the first, except that it was conducted using GAN-generated images. The mean accuracies of the five experts were calculated for each task.

#### 2.2.2 Training CNN classifier

To develop and evaluate the performance of the chest radiograph classifier, four CNN networks were used, namely ResNet50, VGG16, InceptionV3, and DenseNet121. Moreover, we trained SqueezeNet and MobileNetV2 to check the performance of shallow CNN models. Each network was trained on three train/validation sets: the original dataset, the traditionally augmented dataset, and the GAN-augmented dataset. Pre-trained ImageNet models were used to customize the classifiers and for each pre-trained network, the last fully connected layer was modified to match the number of classes (four). The models were trained with a batch size of 16 and a SGD optimizer with a learning rate of 1× (10)^-4^. To avoid overfitting, we enabled the early stopping with patience = 10. The output layers of models includ the softmax activation function and the categorical cross-entropy loss function. The architectures of networks are described in S1 Fig in File. After training, the models were evaluated using a test set with a separate chest radiograph dataset (OpenI). The learning curves of the evaluated CNN classifiers are shown in S2 Fig in S1 File.

#### 2.2.3 Training GAN

StarGANv2 was trained using the Adam optimizer with momentum parameters β_1 = 0 and β_2 = 0.99 at a learning rate of 1×(10)^-4^ for the generator, discriminator, and style encoder, and 1× (10)^-6^ for the mapping network. The total loss function consists of adversarial loss, style reconstruction loss, style diversification loss, and cycle consistency loss to preserve source characteristics. The training phase required approximately 250,000 iterations (141 hours) with a batch size of 8 on a NVIDIA DGX A100×2 (122 GB RAM) GPU.

## 3. Results

**Fig 3:**
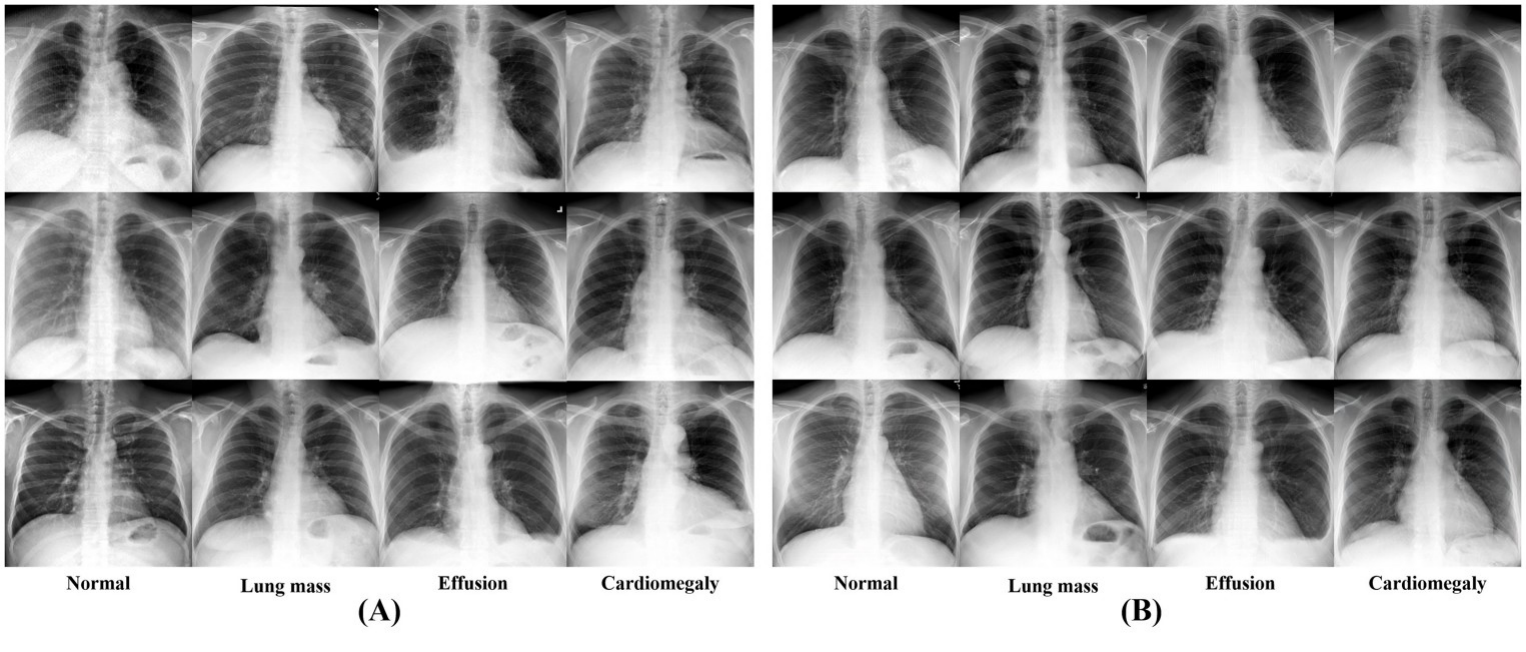
(A) Real images and (B) GAN-generated images.

In this section, an analysis on the quality of the GAN-generated radiographs and whether GAN augmentation enhances the CNN-based classifier performance is presented. The synthetic radiographs generated by the GAN are shown with the actual images in Fig 3.

### 3.1 Visual Turing test

In the visual Turing test, the radiologists were asked to distinguish GAN-generated radiographs from actual images. The mean accuracy of the five radiologists was 66.2%, which was higher than that of random guessing (341/515, 66.2% vs. 257.5/515 50.0%, respectively, P = 0.006). However, in terms of identifying GAN-generated images as artificial, there was no significant difference in the sensitivity of the radiologists and random guessing (147/275, 57.8% vs. 137.5/275, 50.0%, respectively, P = .284). A McNemar test found that only three of the five radiologists (R3, R4, and R5; P = .002, 0.014, <0.001, respectively) performed better than random guessing. The average MCC value was 0.351, ranging from 0.184 to 0.549. The results presented are given in Table 4.

**Table 4:** Visual Turing test result metrics.

	Accuracy (%)	Precision (%)	Recall (%)	F1-score	MCC
**R1**	59.2	60.3	69.1	0.644	0.184
**R2**	77.7	80.8	76.4	0.785	0.549
**R3**	67.0	81.8	49.1	0.614	0.387
**R4**	69.9	81.6	56.4	0.667	0.428
**R5**	57.3	67.7	38.2	0.488	0.208

The radiologists were then given two image sets, one with 103 actual chest radiographs and another with 103 GAN-generated radiographs, and asked to classify each image. Each dataset equally contained three disease classes (cardiomegaly, pleural effusion, lung mass), and one normal class.

Results showed that the experts performed better on GAN-generated images than actual radiographs, (average accuracy 73.7% vs 58.6%, P<0.01), with the mean accuracy differing significantly in larger pathology classes (cardiomegaly and pleural effusion) but not in smaller lesions (lung mass) or normal images. The resulting metrics for the multiclass classification task can be found in Table 5. A possible explanation for this interesting gap in accuracy is presented in the discussion section.

**Table 5:** Classification accuracy by lesion and image type.

	Actual Images	GAN-Generated Images	P-value
Lung Mass	0.385	0.253	0.056
Effusion	0.613	0.808	< .001**
Cardiomegaly	0.483	0.835	< .001**
No Findings	0.887	0.819	0.122
**Average**	0.586	0.737	< .001**

### 3.2 CNN performance

Four CNN-based classifiers, each with a ResNet50, VGG16, InceptionV3, and DenseNet121 backbone, were trained and evaluated. Each classifier was trained on three different train/validation sets, namely the original cumulative train/validation set before augmentation, traditional augmentation (TA), and GAN augmentation. The augmentation amplitudes are presented in Table 3.

The classifiers were then tested on a test set comprising a separate dataset (OpenI) to determine the external validity of the models. The ResNet50 model outperformed the other two classifiers, achieving 83.9% accuracy after GAN augmentation. This model also benefited the most from GAN augmentation, with a 6.6% increase in performance from 78.7% to 83.9%. After GAN augmentation, the accuracy of this model was comparable to that of radiologists. The performance metrics for the multiclass classification are listed in Table 6 and the performance of shallow CNN models could be found in S1 and S2 Tables in S1 File.

**Table 6:** Multiclass classification accuracy performance of each CNN classifier.

	Original	TA	GAN
**ResNet50**	78.7%	77.5%	83.9%
**VGG16**	77.7%	80.1%	82.2%
**InceptionV3**	70.6%	65.2%	75.0%
**DensenNet121**	72.9%	68.7%	79.3%

**Fig 4:**
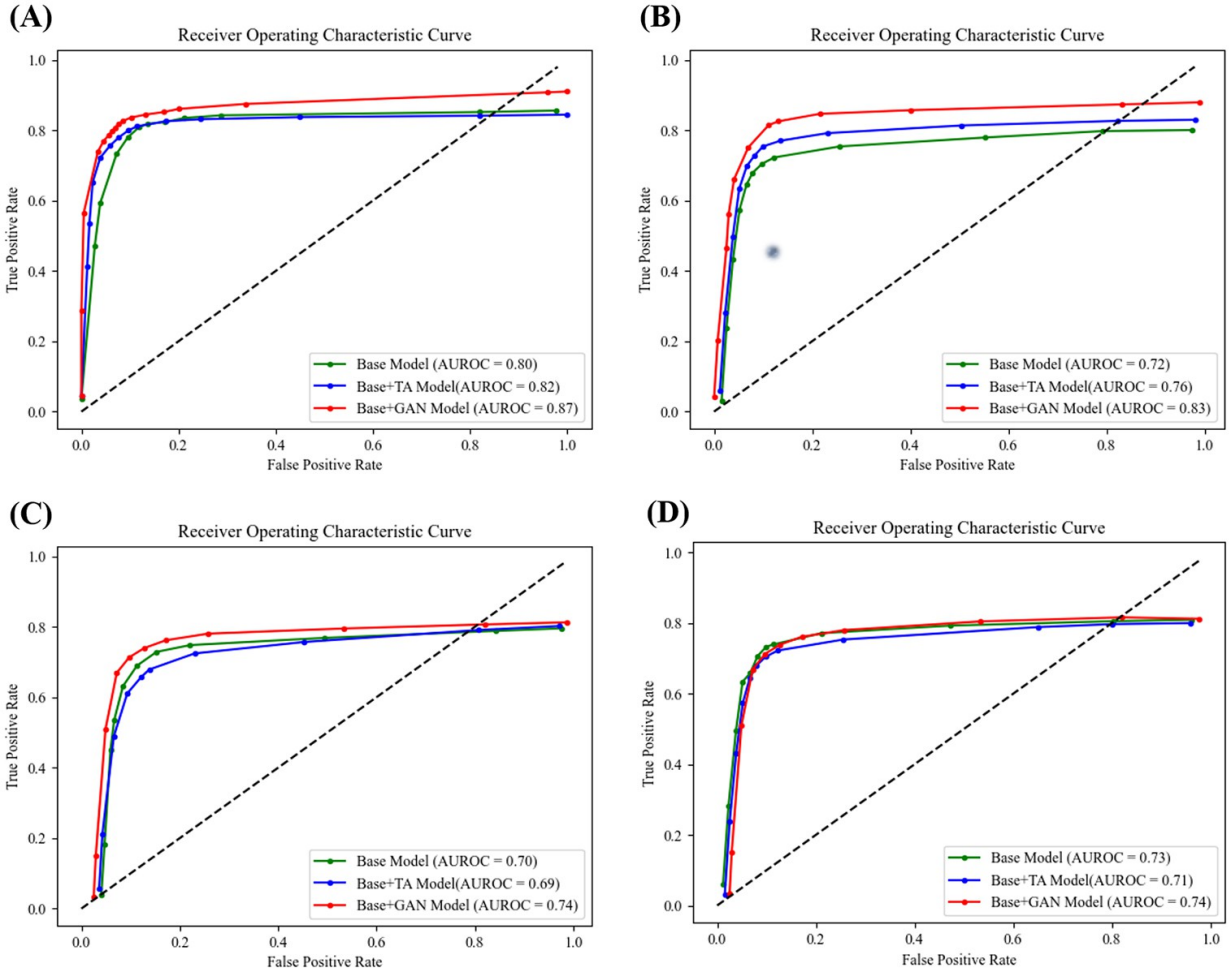
ROC curves of classifiers. (A) ResNet50, (B) VGG16, (C) InceptionV3, and (D) DenseNet121.

In a binary classification task, wherein the classifier determined whether an image was normal (without any pathologic findings) or abnormal (with any pathologic finding), the effect of GAN augmentation was more evident. The ResNet50 model also outperformed the other two classifiers, with an AUROC of 0.871. This model benefited the most from GAN augmentation, increasing its AUROC by 8.6% from 0.802 to 0.871. The performance metrics of binary classification are listed in Table 7 and the receiver operating characteristic (ROC) curves of the ResNet50 model are shown in Fig 4.

**Table 7:** Binary classification performance of each CNN classifier.

		Accuracy	Precision	Recall	F1-score	MCC	AUROC
**ResNet50**	Original	0.845	0.609	0.899	0.726	0.646	0.802
	TA	0.865	0.661	0.842	0.740	0.659	0.823
	GAN	0.901	0.725	0.913	0.808	0.751	0.871
**VGG16**	Original	0.842	0.603	0.910	0.725	0.647	0.717
	TA	0.844	0.600	0.959	0.738	0.671	0.755
	GAN	0.862	0.630	0.962	0.761	0.699	0.828
**InceptionV3**	Original	0.795	0.533	0.828	0.649	0.538	0.697
	TA	0.762	0.488	0.809	0.608	0.482	0.690
	GAN	0.824	0.579	0.850	0.689	0.593	0.741
**DenseNet121**	Original	0.859	0.663	0.781	0.718	0.628	0.726
	TA	0.825	0.569	0.962	0.715	0.644	0.718
	GAN	0.879	0.793	0.637	0.706	0.637	0.741

## 4. Discussion

This study addressed two distinct questions: firstly, whether the generated radiographs deemed realistic to the medical experts, and secondly, whether the GAN-based data augmentation improved the performance of the in-house medical AI.

An open-source, heterogeneous group of chest radiograph datasets was used to ensure that the proposed GAN model was robust to different acquisition parameters of various X-ray machines. As shown in Fig 3, the model produced a realistic repertoire of chest radiographs. Considering that data heterogeneity may result in the production of imperfect chest radiographs, the quality of the generated images was evaluated by asking radiologists working at university hospitals with at least five years of clinical experience to determine whether a given image was real or AI-generated.

As shown in Table 4, radiologists could distinguish GAN-generated images from real images with a 66.2% accuracy. Although this accuracy is better than random guessing, the sensitivity, or the accuracy of correctly telling a GAN-generated image apart, was not significantly different from random guessing. Furthermore, the responses from two of the five radiologists were not significantly different from random guessing, according to the McNemar test. This illustrates that the synthesized images appeared realistic to medical experts.

Subsequently, an evaluation was conducted on the performance of medical experts on multiclass classification of pathologies on both real and GAN-generated images. Here, the average accuracy was 58.6% for actual images and 73.7% for GAN-generated images (Table 5). The apparent gap in performance is perplexing and raises the question of how the radiologists performed better on the AI-generated image set.

As shown in Table 5, the radiologists diagnosed pleural effusion and cardiomegaly significantly better. However, diagnostic performance on lung mass and normal images did not differ significantly across the two groups. This discrepancy may be explained by the lesion size. Lung masses are small masses within the lung parenchyma that are less than 3 cm in size, and pleural effusion is the collection of fluid at the bottom of the thorax, seen as blunted costophrenic angles on radiographs. Cardiomegaly can be detected by the enlargement of heart shadow over 60% of the thoracic width. Cardiomegaly and pleural effusion are both large lesions and present distinct features that GAN can detect. Because the GAN generator learns to replicate distinct features of an image to convince the discriminator that the image is real, large features that strongly represent the real pathology may be reinforced, making the GAN generate radiographs with pathologies easier to classify.

Tables 6 and 7 show that GAN augmentation enhanced CNN classifier performance significantly. This result is noteworthy because it showed that GAN augmentation of existing clinical datasets could improve the medical AI aided diagnostic performance. In all of the CNN classifier models, training on GAN augmented dataset improved medical AI performance. Because CNN based model performances rely on the amount and quality of trainable data, the present result proposes a possibility of improving existing computer aided diagnosis programs. If medical doctors were to use medical AI aided diagnosis algorithms, they would benefit from AI’s that show better performance by training on GAN augmented dataset. Additionally, the shallow CNN models also show increased performance with GAN augmented dataset (S1 and S2 Tables in S1 File), despite the relatively small parameters. These results show that GAN augmentation could have a potentially meaningful role in future lightweight CNN model research.

These findings suggest two distinct strengths of GAN augmentation for chest radiographs. Firstly, GAN-generated images retain most clinical features and are difficult to distinguish from real images, even by clinical experts. Secondly, while GAN-generated images are similar to real images, they also emphasize important clinical attributes, such as pathological radiological signs. While these strengths may serve to enhance the overall performance of medical AI, they can also serve to reproduce medical data with distinct features for human educational purposes. In their 2018 research, Finlayson *et al*. proposed a similar GAN-based training tool for medical education [31]. GAN images may serve as effective augmentation, especially in low-data regimes such as medical fields.

This study has limitations. Firstly, the chest radiographs used were 256 × 256 pixels. In clinical settings, radiologists have access to powerful diagnostic software and images that are at least 2048 × 2048 pixels in size, however, this is only a technical limitation. The resolution of generated radiographs can be expanded with more powerful computing resources. Meanwhile, the medical experts achieved competent classification accuracy even with the current resolution. Another limitation is that the research employed only public-domain datasets. The public datasets employed in this study are prone to data ambiguity because many of them used classification algorithms instead of manual annotation during labeling. Samples from the datasets, however, have been cross-checked by human experts to ensure that they are of competent clinical quality.

This research used GAN to produce chest radiographs that not only improved AI performance but were also convincing as real images to board-certified radiologists. From here, there are plans to extend the research to other imaging modalities with imbalanced datasets, such as rare brain tumors or pediatric cancer.

## 5. Conclusion

The proposed GAN produced realistic chest radiographs that appeared realistic to experienced radiologists; in the visual Turing test, there was no significant difference in the sensitivity of identifying GAN images as artificial between radiologists and random guessing. Radiologists effectively classified chest pathologies with the synthesized radiographs, suggesting that the GAN images contained adequate clinical information for diagnosis. The multi-center visual Turing test found that GAN tends to emphasize larger lesions better than smaller pathologies. To the best of our knowledge, this finding has not been reported before in medical image analysis using GAN. CNN based classifiers rely on the amount and quality of training data, and therefore the result of the present study is noteworthy because it showed that GAN augmentation could effectively augment training data for medical AI. Augmentation through the synthesized images significantly enhanced CNN classifier performance, providing a successful means to overcome data imbalance in medical image analysis.

## Supporting information

S1 File (DOCX)
