# Supplementary material for: Evaluating diagnostic content of AI-generated chest radiography: A multi-center visual Turing test
Source: PLoS One. 2023 Apr 12;18(4):e0279349. doi: 10.1371/journal.pone.0279349 (PMC10096231; doi:10.1371/journal.pone.0279349)
Supplement: S1 File — (DOCX) [file pone.0279349.s001.docx]

**
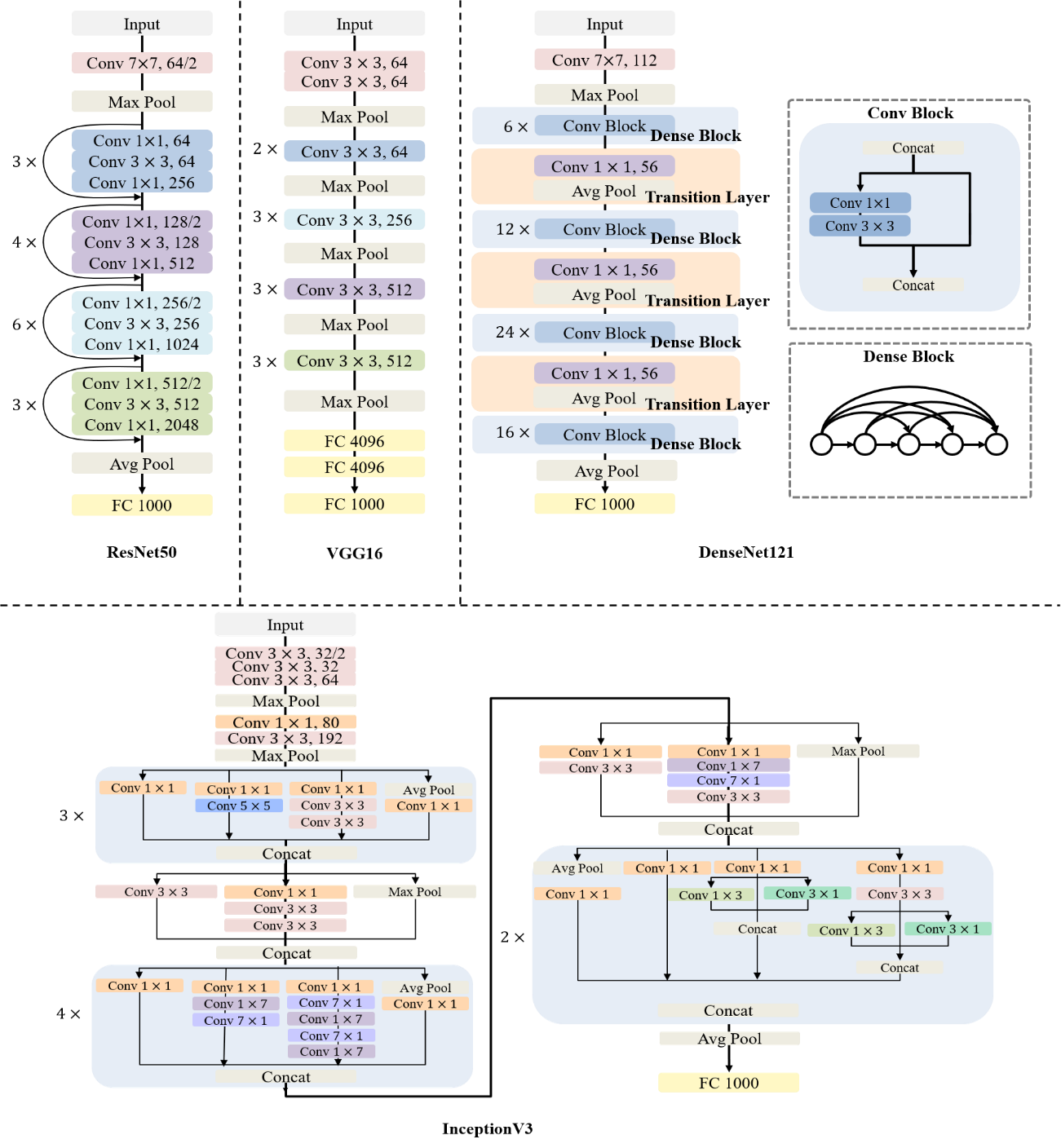
**

**S1 Fig. ResNet50, VGG16, InceptionV3, and DenseNet121 architectures**


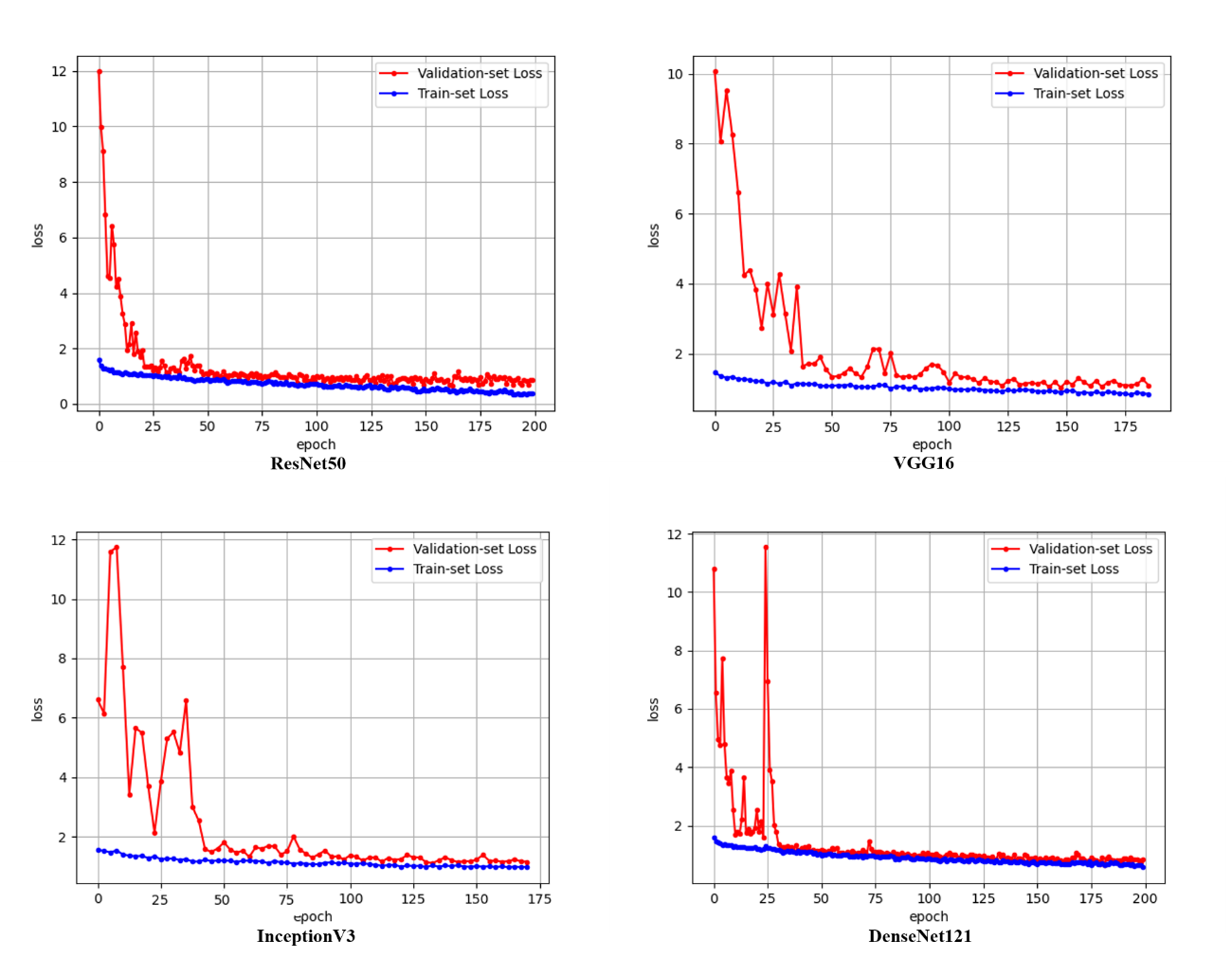


**S2 Fig. Learning curves of ResNet50, VGG16, InceptionV3, and DenseNet121**

**S1 Table. Multiclass classification accuracy performance of shallow CNN models.** TA: traditional augmentation; GAN: generative adversarial networks.

|  | **Original** | **TA** | **GAN** |
| --- | --- | --- | --- |
| **SqueezeNet** | 56.7% | 57.7% | 60.9% |
| **MobileNet** | 60.3% | 56.4% | 68.7% |

**S2 Table. Binary classification performance of shallow CNN models**

|  |  | **Accuracy** | **Precision** | **Recall** | **F1-score** | **MCC** | **AUROC** |
| --- | --- | --- | --- | --- | --- | --- | --- |
| **SqueezeNet** | Original | 0.656 | 0.396 | 0.948 | 0.558 | 0.437 | 0.732 |
|  | TA | 0.614 | 0.367 | 0.943 | 0.528 | 0.392 | 0.696 |
|  | GAN | 0.698 | 0.416 | 0.789 | 0.545 | 0.391 | 0.658 |
| **MobileNet**  **V2** | Original | 0.707 | 0.430 | 0.858 | 0.573 | 0.439 | 0.640 |
|  | TA | 0.706 | 0.428 | 0.841 | 0.568 | 0.429 | 0.611 |
|  | GAN | 0.714 | 0.435 | 0.828 | 0.570 | 0.430 | 0.640 |
